# PSMA-Positive Follicular Thyroid Carcinoma Incidentally Detected by [^68^Ga]Ga-PSMA-11 PET/CT: Correlation with Immunohistology Confirms Neovascular PSMA-Expression

**DOI:** 10.3390/diagnostics12051211

**Published:** 2022-05-12

**Authors:** Florian Rosar, Caroline Burgard, Christian Neubert, Phillip R. Stahl, Fadi Khreish, Samer Ezziddin

**Affiliations:** 1 Department of Nuclear Medicine, Saarland University, 66421 Homburg, Germany; florian.rosar@uks.eu (F.R.); caroline.burgard@uks.eu (C.B.); fadi.khreish@uks.eu (F.K.); 2 Department of Otorhinolaryngology, Head and Neck Surgery, Saarland University, 66421 Homburg, Germany; christian.neubert@uks.eu; 3 Department of Pathology, Saarland University, 66421 Homburg, Germany; phillip.stahl@uks.eu

**Keywords:** PSMA, PET/CT, follicular thyroid cancer

## Abstract

We present an interesting image of an intense PSMA-positive follicular thyroid carcinoma incidentally detected by [^68^Ga]Ga-PSMA-11 PET/CT in a 76-year-old man with biochemical recurrence of prostate cancer. Immunohistochemical staining demonstrated PSMA expression in the endothelial cells of tumor tissue. This interesting image should remind colleagues to consider malignant thyroid neoplasia in PSMA-positive thyroid lesions.

A 76-year-old man presented with biochemical recurrence (BCR) of prostate cancer with an increase in the prostate-specific antigen (PSA) serum value to 1.4 ng/mL (doubling time: approximately 6 months). The man received his diagnosis of prostate cancer 11 years ago (iPSA 4.2 ng/mL, Gleason 4 + 5, pT2c). The patient had undergone prior local therapies, such as robotic prostatectomy, salvage local lymphadenectomy, and salvage external beam radiation of prostate bed (total dose of 66.6 Gy in 37 fractions of 1.8 Gy). For localization of recurrence, we performed a prostate-specific membrane antigen (PSMA)-targeted positron emission tomography–computed tomography (PET/CT) using [^68^Ga]Ga-PSMA-11, which is an established imaging modality in the management of prostate cancer [1,2,3]. [^68^Ga]Ga-PSMA-11 PET/CT revealed no suspicious uptake in the pelvis or retroperitoneal region, but highly suspicious intensive PSMA-positive mass (SUV_max_ 32.2) was detected in the left thyroid (Figure 1). 

In clinical examination, a palpable formation was identified on the left side of the neck. Subsequently, we performed a sonography of thyroid and neck showing an approximately 5 cm large echo-complex nodule in the left thyroid. Blood examination revealed normal TSH, fT3, fT4, and antibody values. Fine needle aspiration was waived and thyroidectomy was performed. Histopathological preparation revealed a minimally invasive follicular thyroid carcinoma with limited capsular penetration (Figure 2A). PSMA-positivity in malignant thyroid neoplasm was also noted by other authors [4,5,6]. It is presumed that PSMA positivity results from frequent PSMA expression in endothelium of tumor microvasculature [7,8,9]. Consistently, we detected PSMA expression predominantly in the endothelial cells of tumor tissue by immunohistochemical staining (Figure 2B). In addition to follicular thyroid carcinoma, PSMA positivity has also been found in other histo-pathological forms of thyroid cancer, such as papillary, poorly differentiated, anaplastic or medullary thyroid carcinoma, as well as in benign thyroid tumors, such as follicular adenoma. However, PSMA positivity seems to be more frequent in malignant tumors, as opposed to benign thyroid tumors [9]. Metastases of prostate cancer to the thyroid gland have also been described in the literature [10,11] and recently demonstrated in PSMA-targeted PET/CT imaging [12]. However, it appears that this localization is extremely rare. Differentiation of entities by [^68^Ga]Ga-PSMA-11 PET/CT appears to be very difficult; thus, histologic or cytologic examination should be sought. 

This interesting image should remind colleagues to consider malignant thyroid neoplasia in PSMA-positive thyroid lesions.

## Figures and Tables

**Figure 1:**
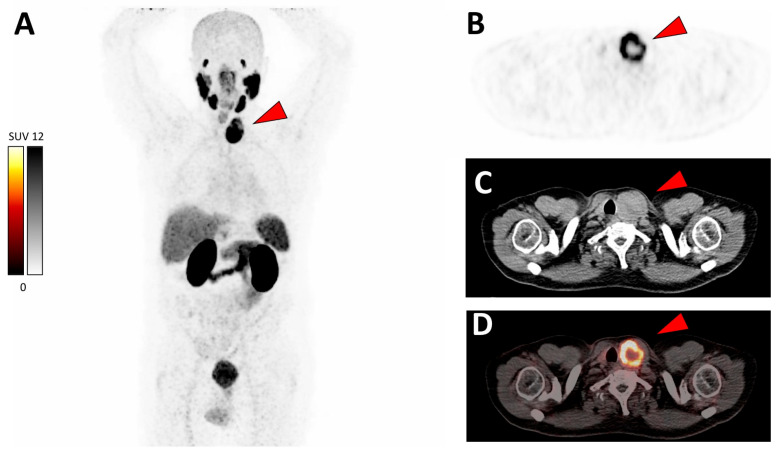
[^68^Ga]Ga-PSMA-11 PET/CT showing intense uptake (SUV_max_ 32.2) in a left thyroid mass. (**A**) Maximum intensity projection (MIP); transversal slices of (**B**) PET, (**C**) CT, and (**D**) PET/CT fusion. Red arrows point to the left thyroid mass.

**Figure 2:**
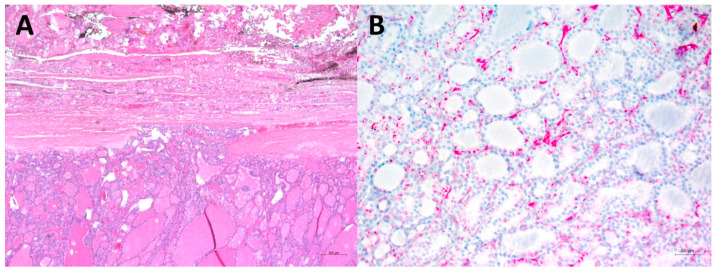
Histopathologic images showing minimally invasive follicular thyroid carcinoma. (**A**) Limited capsular penetration by the follicular neoplasia; hematoxilin–eosin (H&E) stain, magnification 50×. (**B**) Immunohistochemical analysis for PSMA: endothelial cells showed strong positivity in several small interfollicular vessels, magnification 200×.

## Data Availability

The datasets used and analyzed in this paper are available from the corresponding author on reasonable request.
